# Deep carbon cycling during subduction revealed by coexisting diamond-methane-magnesite in peridotite

**DOI:** 10.1093/nsr/nwad203

**Published:** 2023-07-24

**Authors:** Xiaoxia Wang, Yilin Xiao, Hans-Peter Schertl, Nikolay V Sobolev, Yang-Yang Wang, He Sun, Deshi Jin, Dong-Bo Tan

**Affiliations:** CAS Key Laboratory of Crust-Mantle Materials and Environments, School of Earth and Space Sciences, University of Science and Technology of China, China; CAS Key Laboratory of Crust-Mantle Materials and Environments, School of Earth and Space Sciences, University of Science and Technology of China, China; CAS Center for Excellence in Comparative Planetology, China; Ruhr-University Bochum, Faculty of Geosciences, Institute of Geology, Mineralogy and Geophysics, Germany; V.S. Sobolev Institute of Geology and Mineralogy, Siberian Branch of Russian Academy of Sciences, Russia; CAS Key Laboratory of Crust-Mantle Materials and Environments, School of Earth and Space Sciences, University of Science and Technology of China, China; School of Resources and Environmental Engineering, Hefei University of Technology, China; CAS Key Laboratory of Crust-Mantle Materials and Environments, School of Earth and Space Sciences, University of Science and Technology of China, China; CAS Key Laboratory of Crust-Mantle Materials and Environments, School of Earth and Space Sciences, University of Science and Technology of China, China

## Abstract

Identification of multiphase inclusions in peridotite suggests that released carbon from a subducting slab can be stored as diamond+methane+magnesite in the overlying mantle wedge, achieving deep carbon cycling.

Subduction is an important process for transferring materials from the surface into the deep mantle. During subduction, recycled carbon can be released from the subducting slab to the mantle wedge through various decarbonation processes. Both oxidized (CO_2_/CO_3_^2^^−^) and reduced (CH_4_) carbon-bearing species have been discovered in (ultra)high-pressure (HP-UHP) subduction zones worldwide [1–3]. Diamond is a typical index mineral for UHP metamorphism. Stable isotope studies on these diamonds have demonstrated that recycled crustal carbon contributes to their formation, indicating that diamonds represent a powerful phase in deep carbon cycling. The presence of syngenetic carbonate, sulfate, and hydrocarbon inclusions indicates that oxygen fugacity plays an important role in the formation of such diamonds [4,5]. Furthermore, relictic carbon phases coexisting with diamond are key sources for diamond crystallization. As a result, carbonate reduction and methane oxidation are important mechanisms for diamond formation [3,4]. However, the redox environment present during diamond crystallization and the precise genetic relationship between diamond, methane, and carbonates are poorly constrained.

Oxidizing carbon in CHO fluids is in equilibrium with carbonates at shallow depths in the subduction zone, whereas it decomposes to form magnesite + calcite + CH_4_ + C [6] or C + O_2_ [5] under reducing and oxidizing conditions, respectively, through metasomatic processes. Consequently, as the depth increases, oxidizing carbon species transform into stable diamond/graphite. Recently, oxygen fugacity was shown to be reduced at sub-arc depths; thus, reduced carbon species are likely the major carbon compounds [6]. Abundant reduced abiotic CH_4_ has also been detected in subduction zones [4,6]. However, the stable carbon-bearing species that occur where diamond is stable remain under debate. Such controversy hampers our understanding of carbon migration in subduction zones beyond sub-arc depths, making it difficult to estimate the global carbon flux and its contribution to deep carbon cycling.

The CHO fluids released during subduction can react with the overlying mantle wedge as recorded by inclusions. Carbon phase inclusions in orogenic peridotite could corroborate carbon migration during upward infiltration, representing an ideal agent for tracing the role of CHO fluids. Thus, a diamondiferous mantle wedge-type (M-type) peridotite experiencing complex CHO fluid-rock interaction with the subducting slab can provide key insights into diamond formation and carbon cycling.

The Triassic Dabie-Sulu orogenic belt in China is one of the largest exposed UHP terrains [1]. Within olivine of M-type Raobazhai peridotite in the Dabie orogenic belt, we found coexisting micro-diamond, methane, and magnesite. We demonstrated that the three carbon phases were likely stable at depths where diamond is stable. This is important for understanding the characteristics of subduction-related diamond formation, the relationship between the involved carbon phases, and their implications for deep carbon cycling.

The studied peridotite belongs to a segment of the overlying mantle wedge of the Dabie orogenic belt (Fig. S1). Furthermore, the peridotite enclosed eclogite forming lense-like bodies. The mineral assemblage of the peridotite is olivine + orthopyroxene + clinopyroxene + amphibole + spinel + serpentine + zircon (Fig. S2A–B); olivine grains contain abundant randomly distributed inclusions (Fig. S2C–D). Using a newly developed 3D Raman tomographic microspectroscopy analysis, we confirmed the coexistence of micro-diamonds with methane and magnesite in an inclusion (Video S1, Fig. 1) indicating simultaneous growth. In addition, the studied diamonds are characterized by sp^3^ (1334 cm^−1^) and sp^2^ (1568 cm^−1^) carbons, slightly shifting from the value of diamond abrasives (1332 cm^−1^) (Figs S3 and S4). Abundant methane (2917 cm^−1^) and magnesite (1094 cm^−1^ and 329 cm^−1^) inclusions were also detected in olivine (Fig. S3). In addition, high-salinity fluid inclusions were identified by Linkam THMSG600 heating/freezing equipment, with a freezing temperature of approximately −41°C. This indicated that the fluid inclusions contained dissolved ions.

**Figure 1.**
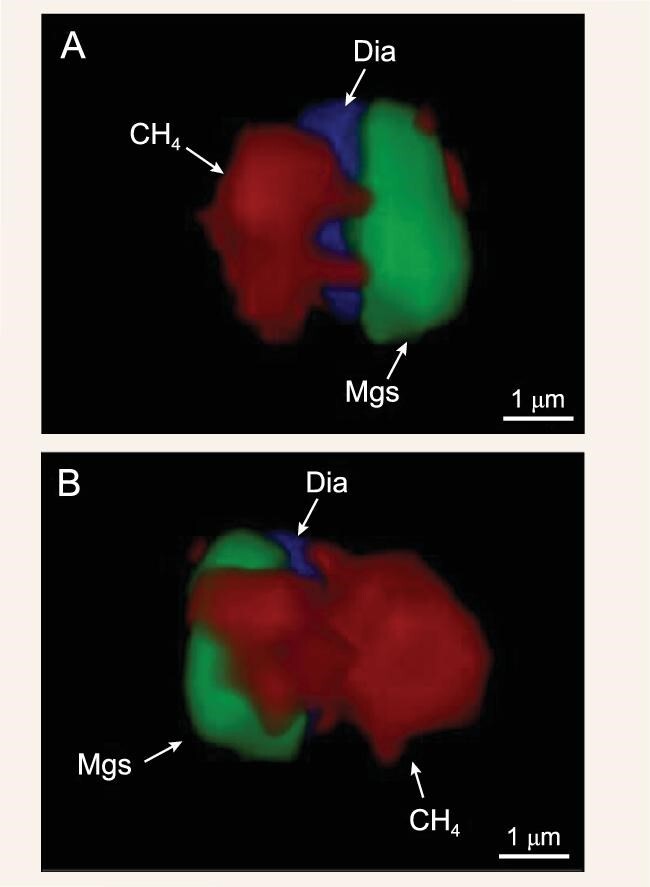
A, B: Three-dimensional multiphase inclusions of Dia (blue), CH_4_ (red), and Mgs (green) in olivine.

The occurrence of a homogeneous luminescent zircon demonstrates that the diamond (Fig. S2E–H) is located below the polished surface of zircon and forms a true inclusion that cannot be sourced from a possible abrasive. The diamond inclusions were identified by SEM-Raman analyses when they still were enveloped by zircon, only further polishing brought them to the surface (Fig. S3D). The inclusion was characterized by bands at 1340 cm^−1^ and 1608 cm^−1^. The G band (1580 cm^−1^) is upshifted to 1608 cm^−1^; thus, the related graphite structure was likely affected by the present defects. In summary, the above data demonstrate true UHP metamorphic inclusions enveloped by olivine and zircon.

Carbon species stored in UHP rock provide important information, contributing to our understanding of deep carbon cycling [4]. In this study, we identified associated diamond, magnesite, and methane forming multiphase inclusions in M-type peridotite (Video S1, Fig. 1). Therefore, carbon-saturated metamorphic fluids were entrapped by olivine before it exhumated to the surface along with the subducted slab. Most carbon released from the subducting slab experiences volcanic processes, whereas the surviving carbon is embedded as carbonate in the down going slab or as an integral part of the migrating metamorphic fluid/melt at great depths. Furthermore, Stagno *et al.* [7] demonstrated that carbonates can transform into a more stable refractory diamond/graphite when the carbonate-bearing fluid/melt migrates to the overlying mantle wedge. However, carbonate reduction/decarbonation occurs in subduction zones under reducing conditions, and carbonate can act as an ideal abiogenic CH_4_ precursor [6,8,9]. Calcite and siderite were detected in garnet from the Raobazhai eclogite (Fig. S6), indicating they were the major carbonate species during carbon transportation in CHO fluids. With slab subduction, siderite reacts with H_2_O (FeCO_3_ in siderite + H_2_O → magnesite + magnetite + CH_4_ + C (diamond/graphite) Equation (1)), resulting in the formation of multiphase Mgs + CH_4_ + Dia inclusion and a decrease in the H_2_O content. As discussed by Tao *et al.* [6], H_2_ is one of the major intermediate products of Equation (1). Although most of the H_2_ was released, the remaining H_2_ reduced the contiguous C(diamond/graphite) through C + 2H_2_ = CH_4_. This is supported by the abundant Mgs + CH_4_ inclusions but rare occurrence of Dia + Mgs + CH_4_ multiphase inclusions in olivine. This is the first case of a diamond-CH_4_ association under reducing conditions. Experimental studies have suggested that hydrogen plays an important role in C−H bond formation during diamond crystallization. Consequently, the diamonds may contain sp^2^ hybrid orbitals in addition to sp^3^ ones. Based on the above studies, the coexisting magnesite-methane-diamond species in the Raobazhai peridotite likely represent an assemblage of stable carbon phases in the mantle wedge under reduced conditions.

With H_2_O consumption through siderite decomposition (reaction (1)), the H_2_O content decreases during subduction, resulting in the formation of high-salinity fluids. In contrast, Pruteanu *et al.* [10] suggested that CH_4_ became miscible in water with increasing pressure. The solubility of CH_4_ decreased in CH_4_-H_2_O fluids when the slab was exhumed. As a result, CH_4_-rich and H_2_O-rich aqueous fluid inclusions rather than CH_4_-H_2_O aqueous fluid inclusion were detected in olivine (Fig. S2C–D, Fig. S3B). Our study shows that the carbon released from a subducting slab can be stored as diamond + methane + magnesite in the overlying mantle wedge. The oxidization transforms Fe-carbonate into CH_4_, magnesite, and refractory diamond at sub-arc depth, achieving carbon cycling at great depths.

## Supplementary Material

nwad203_Supplemental_Files

## References

[1] Xu ST , Okay AI, Ji SY et al. Science 1992; 256: 80–2. 10.1126/science.256.5053.80 17802596

[2] Yang J , Wu W, Lian D et al. Nat Rev Earth Environ 2021; 2: 198–212. 10.1038/s43017-020-00138-4

[3] Luth RW , Stachel T. Contrib Mineral Petrol 2014; 168: 1083. 10.1007/s00410-014-1083-6

[4] Frezzotti ML . Nat Commun 2019; 10: 4952. 10.1038/s41467-019-12984-y 31666507 PMC6821813

[5] Frezzotti ML , Selverstone J, Sharp ZD et al. Nat Geosci 4: 703–6. 10.1038/ngeo1246

[6] Tao RB , Zhang L, Tian M et al. Geochim Cosmochim Acta 2018; 239: 390–408. 10.1016/j.gca.2018.08.008

[7] Stagno V , Frost DJ, Mccammon CA et al. Contrib Mineral Petrol 2015; 169: 16. 10.1007/s00410-015-1111-1

[8] Vitale Brovarone A , Martinez I, Elmaleh A et al. Nat Commun 2017; 8: 14134. 10.1038/ncomms14134 28223715 PMC5322563

[9] Zhang LJ , Zhang LF, Tang M et al. Natl Sci Rev 2023; 10: nwac207 10.1093/nsr/nwac207. 10.1093/nsr/nwac207 36654916 PMC9840456

[10] Pruteanu CG , Ackland GJ, Poon WCK et al. Sci Adv 2017; 3: e1700240. 10.1126/sciadv.1700240 28845447 PMC5567757

