# Supplementary material for: Deep carbon cycling during subduction revealed by coexisting diamond-methane-magnesite in peridotite
Source: Natl Sci Rev. 2023 Jul 24;10(10):nwad203. doi: 10.1093/nsr/nwad203 (PMC10476885; doi:10.1093/nsr/nwad203)
Supplement: nwad203_Supplemental_Files [file nwad203_supplemental_files.zip › method.docx]

**Method**

**Scanning Electron microscope (SEM)-Raman analysis**

The morphology of the diamond inclusions in zircon were analysis by HITACHI SU8220 field emission scanning electron microscope (FESEM) at University of Science and Technology of China (USTC), coupled with energy-dispersive X ray (EDX) on Pt-coated sample. The accelerating potential was 3 kV with working distance of 10mm. The counting time was 100s.

In situ individual inclusion composition analysis was performed by using HORIBA Jobin-Yvon LabRAM HR Evolution confocal Raman microspectrometer, equipped with a laser wave length of 532 nm and a confocal optical, Air-cooled CCD detector at USTC. The Raman analyses were calibrated by the 520 cm^-1^ pure silicate bond.

Three dimensional Raman imaging of multiphase inclusion were carried out on WITec alpha300R confocal Raman microscopy (WITec GmbH) with 532 nm diode-pumped solid-state excitation laser at WiTec company, Beijing. Two dimensional imaging analysis of in situ diamond inclusion composition was performed by SEM-Raman analysis at Institute of Geology and Geophysics, Chinese Academy of Science. WiTec alpha300 confocal Raman microscopy with a 532 nm wavelength laser was selected. Operating power was 0.5 mW for diamond inclusion below the surface of olivine for three dimensional analyses and 0.1 mW for mapping image analysis of diamond inclusion. Finally, the Raman data analyses were performed with WITec Progect plus software.
