# Supplementary material for: Deep carbon cycling during subduction revealed by coexisting diamond-methane-magnesite in peridotite
Source: Natl Sci Rev. 2023 Jul 24;10(10):nwad203. doi: 10.1093/nsr/nwad203 (PMC10476885; doi:10.1093/nsr/nwad203)
Supplement: nwad203_Supplemental_Files [file nwad203_supplemental_files.zip › SFigure+captions.pdf]

SFig. 1

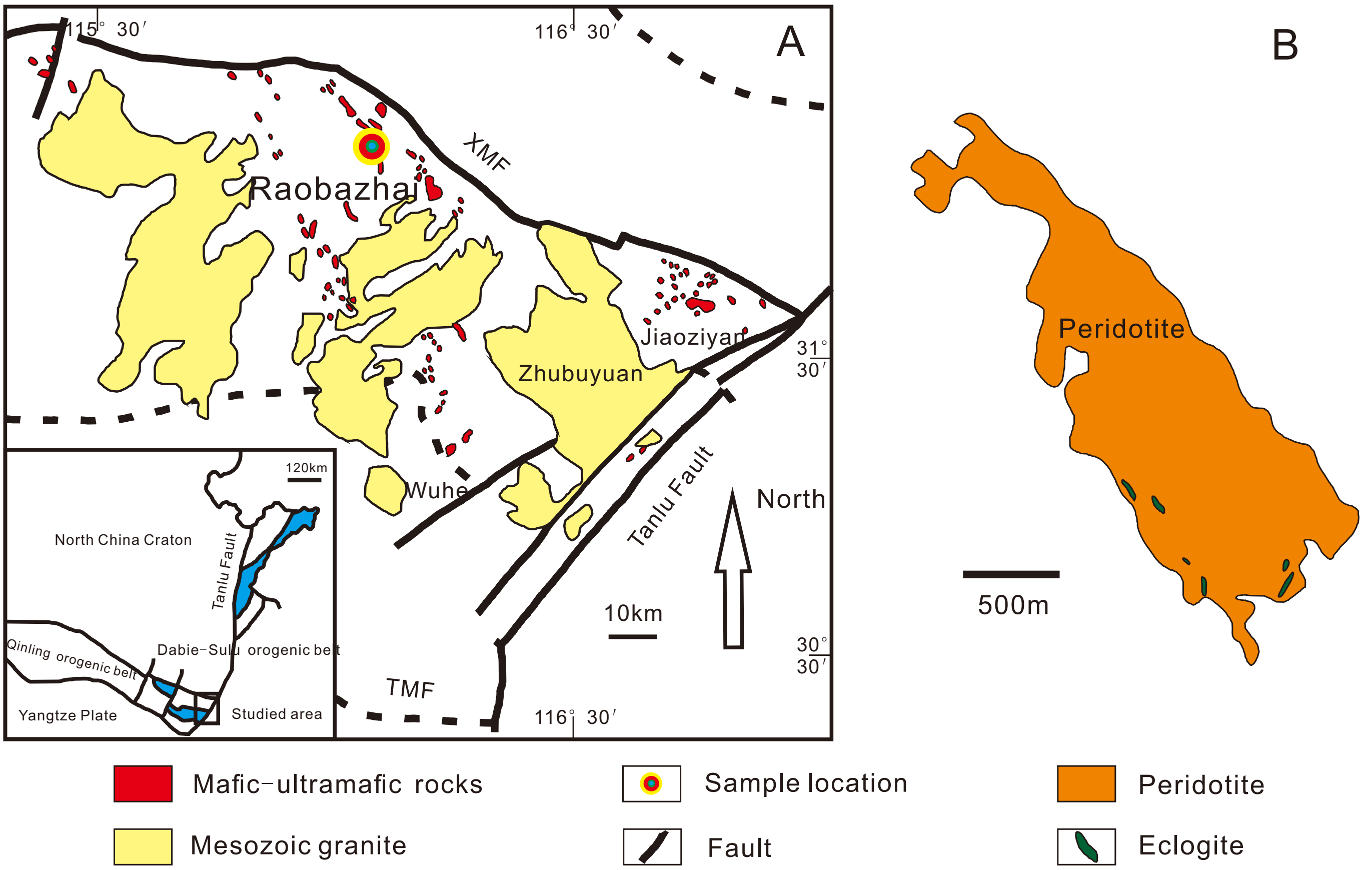
A
115° 30′
116° 30′
XMF
Raobazhai
Jiaoziyan
Zhubuyuan
31° 30′
Wuhe
Tanlu Fault
North
10km
30° 30′
TMF
116° 30′
120km
North China Craton
Tanlu Fault
Dabie–Sulu orogenic belt
Qinling orogenic belt
Yangtze Plate
Studied area
B
Peridotite
500m
Mafic–ultramafic rocks
Mesozoic granite
Sample location
Fault
Peridotite
Eclogite

SFig. 2

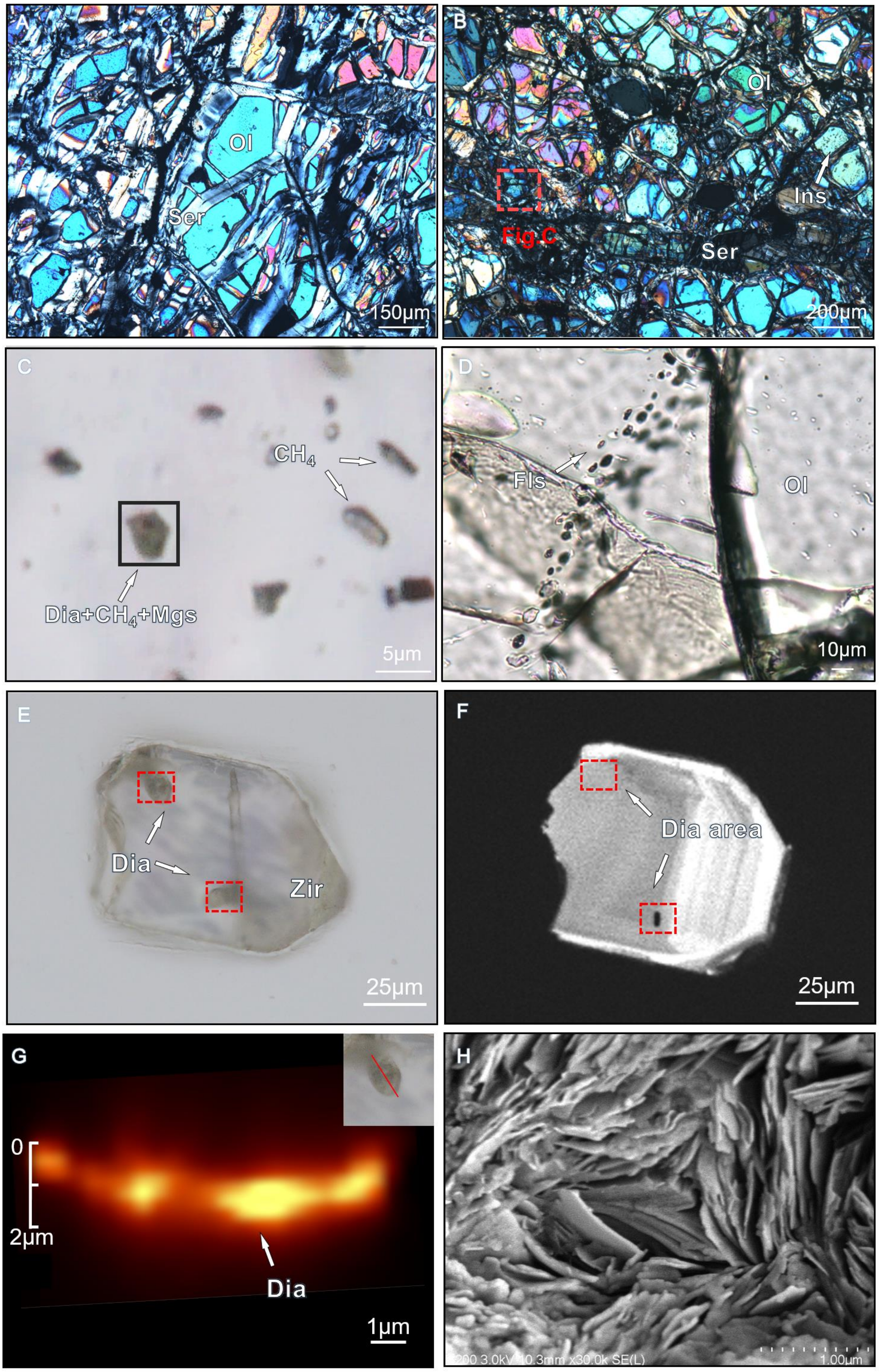

A
Ol
Ser
150μm
B
Ol
Ins
Fig.C
Ser
200μm
C
CH4
Dia+CH4+Mgs
5μm
D
Fls
Ol
10μm
E
Dia
Zir
25μm
F
Dia area
25μm
G
0
2μm
Dia
1μm
H
200 3.0kV 10.3mm x30.0k SE(L)
1.00μm

SFig. 3

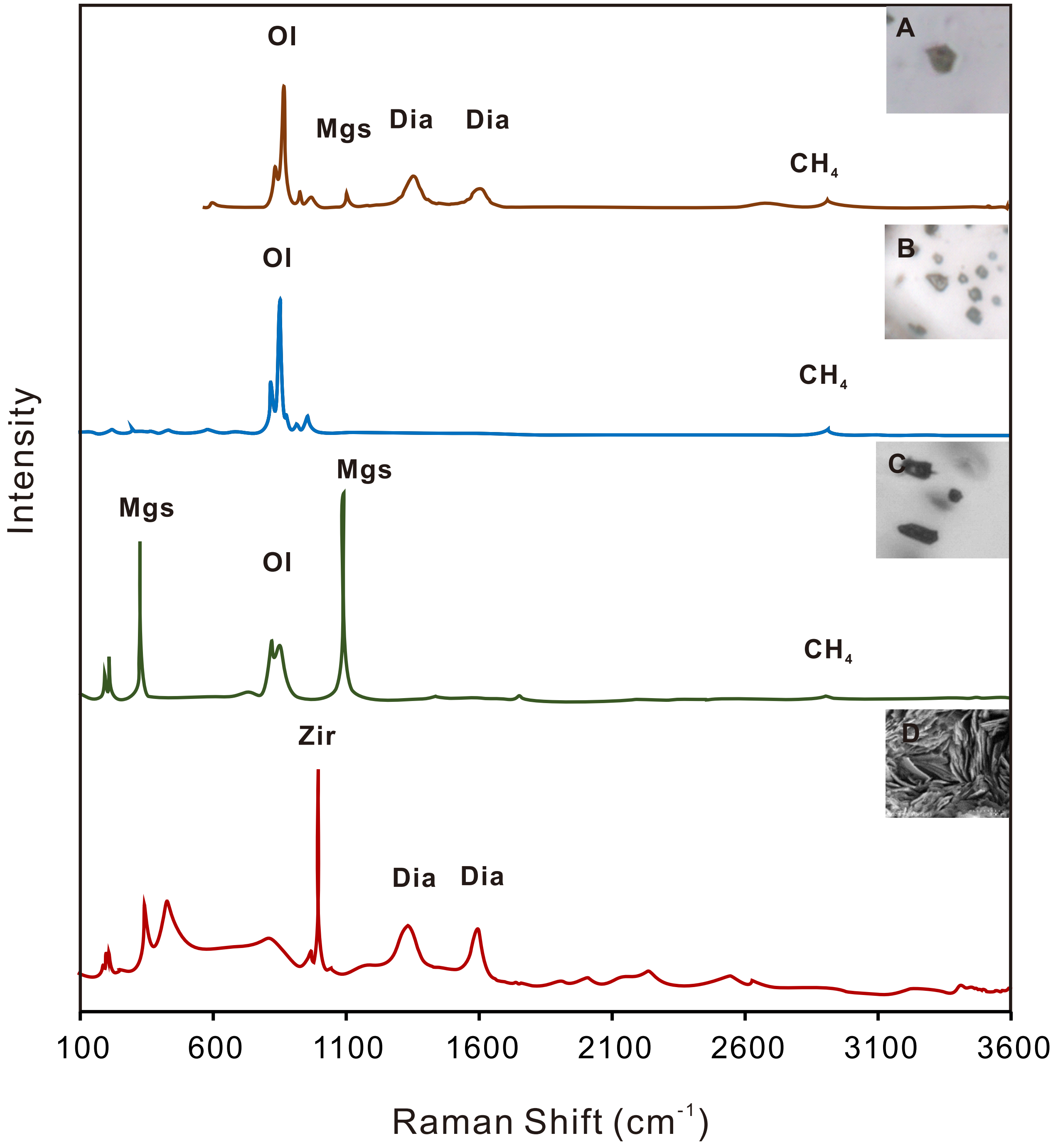

A
B
C
D
Ol
Mgs
Dia
Dia
CH4
Ol
CH4
Mgs
Mgs
Ol
CH4
Zir
Dia
Dia
Intensity
100
600
1100
1600
2100
2600
3100
3600
Raman Shift (cm-1)

SFig. 4

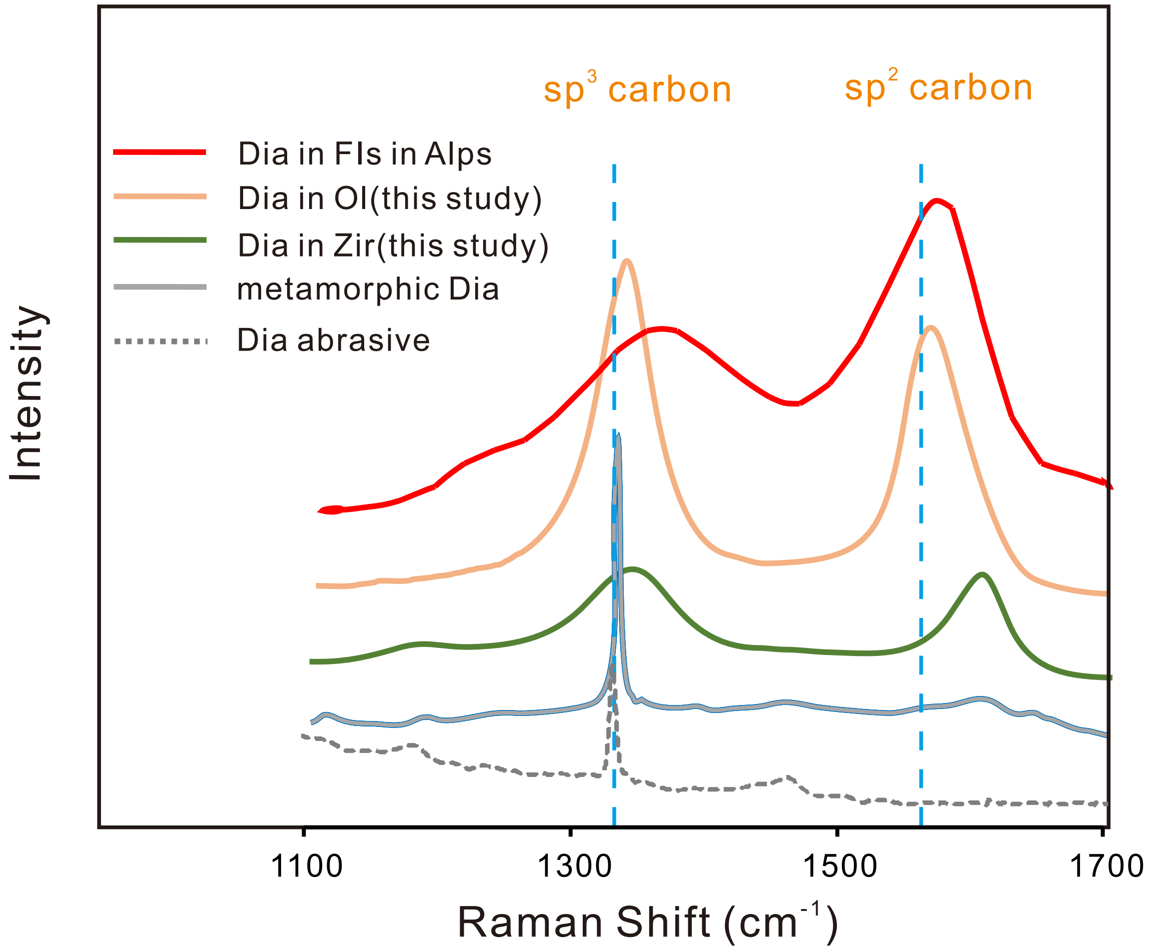
sp3 carbon
sp2 carbon
Dia in FIs in Alps
Dia in Ol(this study)
Dia in Zir(this study)
metamorphic Dia
Dia abrasive
Intensity
1100
1300
1500
1700
Raman Shift (cm-1)

SFig. 5

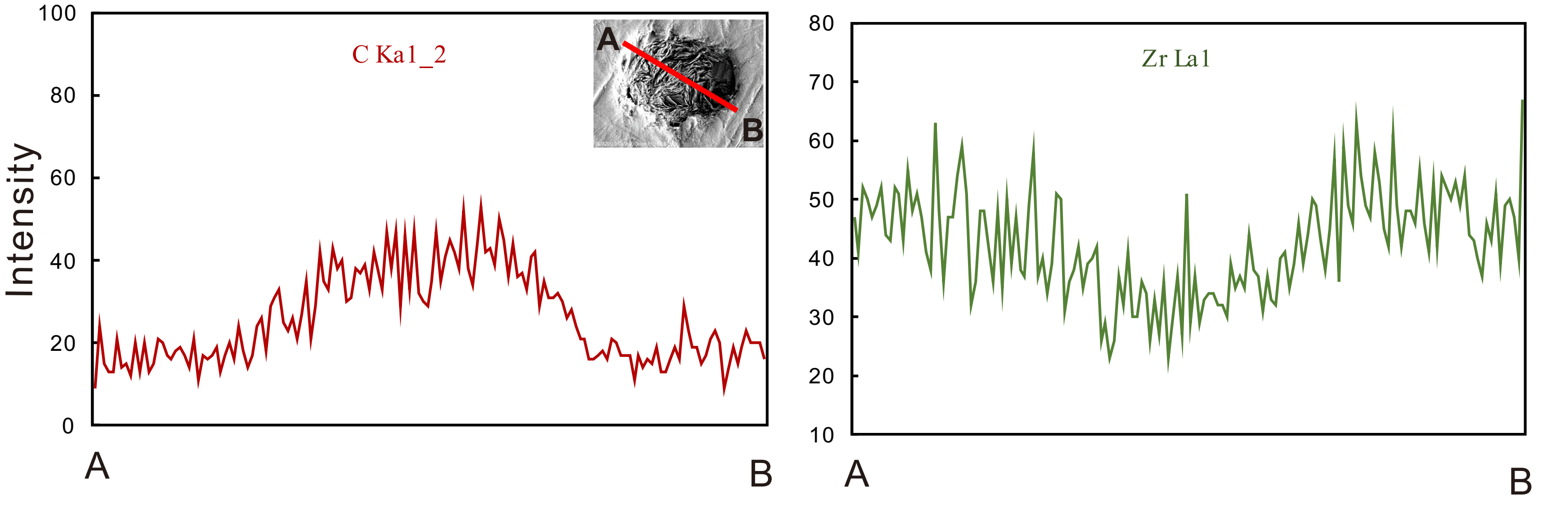
100
80
60
40
20
0
Intensity
C Ka1_2
A
B
A
B
80
70
60
50
40
30
20
10
Zr La1
A
B

SFig. 6

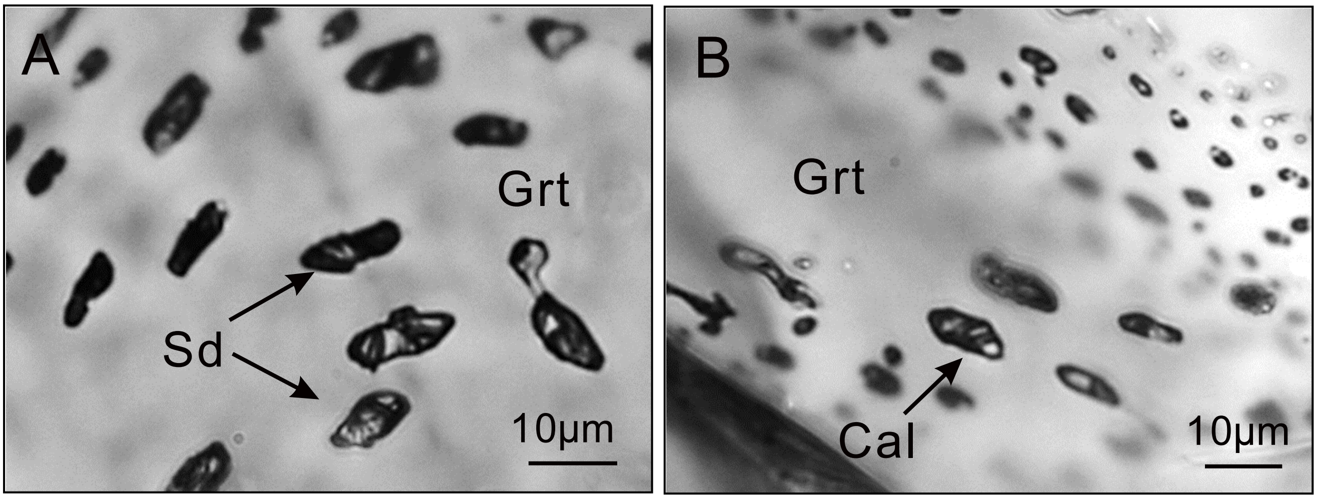

Figure caption:

SFig.1 A: simplified geological map of Dabie orogenic belt (modified after Xiao et al., 2001); B: simplified geological map of geological map of the Raobazhai ultramafic massif (modified after Tsai et al., 2000).

SFig.2 A, B: Transmitted image of diamond-bearing peridotite; C: diamond, methane and magnesite inclusions in olivine; D: secondary high-salinity fluid inclusions in olivine; E: Transmitted image of diamond in zircon; F: CL image of diamond-bearing zircon where diamond was trapped below the surface of 16μm; G: Vertical section Raman image of diamond inclusion which was polished to surface at the position of 0 μm; H: Scanning Electron Microscopy image of diamond in zircon. Dia stands for diamond, Ol stands for olivine, CH_4 stands for methane, Mgs stands for magnesite, FIs stands for fluid inclusions, Zir stands for zircon. The dashed box area represents diamond-bearing olivine grains. The solid box area represents diamond+CH_4+magnesite inclusion.

SFig.3 Raman spectra of A: multiphase inclusion, including diamond, methane and magnesite; B: methane inclusion; C: multiphase inclusion, including magnesite and methane in olivine; D: diamond inclusion in zircon.

SFig.4 Representative Raman spectra of diamond grains, including diamond inclusion in olivine and zircon from Raobazhai, metamorphic diamond from Kokchetav (Sobolev and Shatsky, 1990), diamond in fluid inclusion (FI) from Alps (Frezzotti, 2019) and synthetic diamond abrasive.

SFig.5 SEM-EDS element line section composition of polished diamond, a is carbon element line section; b is zirconium element line section.

SFig.6A: siderite inclusion in garnet; B: calcite inclusion in garnet from Raobazhai eclogite.

Reference

1. Frezzotti M. L, Diamond growth from organic compounds in hydrous fluids deep within the Earth. Nature Communications. 2019. 10(1): 4952.
2. Sobolev N. V, and Shatsky V. S, Diamond inclusions in garnets from metamorphic rocks: a new environment for diamond formation. Nature. 1990. 343(6260): 742-4.
3. Tsai C. H, Liou J. G and Ernst W. G, Petrological characterization and tectonic significance of retrogressed garnet peridotites, Raobazhai area, North Dabie Complex, eastcentral China. Journal of Metamorphic Geology. 2000. 18(2). 181-12.
4. Xiao Y. L, Hoefs J, Kerkhof A, Li S. G, Geochemical constrains of the eclogite and granulite faces metamorphis as recognized in the Raobazhai complex from North Dabie Shan, China. Journal of Metamorphic Geology. 2001. 19(1). 3-17.
